# Childhood Maltreatment, Psychopathology, and Offending Behavior in Patients With Schizophrenia: A Latent Class Analysis Evidencing Disparities in Inpatient Treatment Outcome

**DOI:** 10.3389/fpsyt.2021.612322

**Published:** 2021-01-28

**Authors:** Steffen Lau, Johannes Kirchebner, Sabine Kling, Sebastian Euler, Moritz Philipp Günther

**Affiliations:** ^1^Department of Forensic Psychiatry, University Hospital of Psychiatry Zurich, Zurich, Switzerland; ^2^Computer Vision Laboratory, Department of Information Technology and Electrical Engineering, Swiss Federal Institute of Technology (ETH) Zurich, Zurich, Switzerland; ^3^Department of Consultation Psychiatry and Psychosomatics, University Hospital of Zurich, Zurich, Switzerland

**Keywords:** latent class analysis, schizophrenia, childhood maltreatment, violence, offending behavior, trauma

## Abstract

**Background:** Extant research has provided evidence for disparities between patients with schizophrenia spectrum disorder (SSD) who have and have not experienced childhood maltreatment (CM) in terms of treatment outcome, psychopathology and their propensity to engage in offending behavior. However, research addressing all phenomena is scarce.

**Objective:** The current study aims to explore differences between offender patients with SSD and CM and those with SSD and no CM in terms of their offending, psychopathology at different points in time and treatment outcome.

**Method:** In the present explorative study, latent class analysis was used to analyze differences between 197 offender patients with SSD and CM and 173 offender patients with SSD and no CM, who were admitted to forensic psychiatric inpatient treatment between 1982 and 2016 in Switzerland.

**Results:** Three distinct homogenous classes of patients were identified, two of which were probable to have experienced significant CM. One third of patients with SSD and CM were probable to benefit from inpatient treatment, even surpassing results observable in the group without CM, whereas the other group with SSD and CM was probable to benefit less. Patients with SSD and no CM displayed more psychopathology at first diagnosis and prior to their index offense. Interclass differences in offending behavior were minimal.

**Conclusions:** Offender patients with SSD and CM differ not only from offender patients with SSD and no CM, but also amongst themselves. While some with SSD and CM experience a remission in psychopathology and improve their prognosis for future offending behavior, others do not. Directions for future research on SSD and CM are discussed.

## Introduction

Patients with schizophrenia spectrum disorders (SSD) and offending behavior are a significant population in forensic psychiatry. Evidence suggests that the rate of offending behavior (i.e., illegal violent and non-violent aggression against others) may be twice as high in patients with SSD, in comparison to the overall population, even if age, substance use and socio-economic factors are controlled for (1–3). Furthermore, offending behavior is nine times higher in patients with SSD and substance use as a comorbidity and 19.5 times as high for homicide or attempted homicide (1).

Among the numerous studies on the possible underlying reasons for this association, childhood maltreatment (CM) has been recurrently identified to at least double the odds for both, substance use and offending behavior in patients with SSD (4). CM includes sexual abuse (i.e., sexual contact between child and adult), physical abuse (i.e., bodily assault), emotional abuse (i.e., verbal assaults, humiliation), emotional neglect (i.e., failure of caregivers to provide for basic emotional and psychological needs) and physical neglect (i.e., failure of caregivers to provide for basic bodily needs such as food or shelter) (5–8). While CM might be seen as an unspecific risk factor for various psychiatric diseases (9, 10), some evidence revealed a link between SSD and specific types of CM such as emotional abuse (6, 8) or emotional neglect (7, 9, 10) compared to other psychiatric diseases. Similarly, some research has linked physical abuse to homicide (11) and offending in patients with SSD (12).

Further research revealed that specific types of CM are linked to psychopathology in SSD without including the role of offending behavior. For example, drawing on network analysis, all 5 scales of the Childhood Trauma Questionnaire-Short Form were linked to general psychopathology defined in the Positive and Negative Syndrome Scale (PANSS), which again linked to positive and negative symptoms in the PANSS (7). This means, for instance, Isvoranu et al. linked physical abuse to grandiosity, excitement and hostility via poor impulse control (7). Similarly, physical abuse was connected to hallucinations, delusions and paranoia via somatic concern and unusual thought content. Finally, in the same study, physical neglect was linked to blunted affect, lack of spontaneity and flow of conversation via motor retardation. Such results seem particularly relevant for efforts aiming to optimize treatment outcomes. Especially, since another recent systematic review and meta-analysis reported treatment outcomes to be poorer in SSD when there was a history of CM (13). Odds ratios for poorer treatment outcomes in patients with CM and SSD ranged between 2.35 [CI 1.05–5.27; (14)] and 6.80 [CI 1.66–27.86; (15)].

In summary, studies have shown that specific types of CM might play an important role for offending behavior and psychopathology in SSD. However, the (inter-)relations between CM, offending behavior, and psychopathology in SSD are not sufficiently clear. A more comprehensive analysis, which enlightens the complex interaction between subtypes of CM, psychopathology, and offending behavior in SSD, was recommended in a recent review and meta-analysis on the topic (4). This might allow more targeted preventive efforts of CM, SSD and offending behavior. As suggested by another recent meta-analysis indicating that patients with psychotic disorders and CM (regardless of violent behavior) experience poorer treatment outcomes than patients with psychosis alone (13), the effect of CM on treatment of SSD should be further evaluated in a naturalistic clinical setting in light of the presence or absence of CM.

The present study aims to identify subgroups of patients with SSD based on treatment outcome and the presence of various patient characteristics, such as the specific types of CM, psychopathology, and types of violent and non-violent offending. We were particularly interested in whether or not patients with offending behavior and SSD with and without CM are distinct groups. Further, using an exploratory approach, we investigated whether a particular subtype of CM is more prone to more severe offending or poorer treatment outcome in SSD. Using latent class analysis (LCA), all given variables were explored without prior preconceptions (i.e., preconceived classification of variables into dependent and independent variables). This allows for a more explorative and less biased interpretation of the data set.

## Materials and Methods

### Source of Data and Preliminary Processing

Data on CM, symptoms of SSD and criminal behavior were collected from 370 offender patients with SSD in inpatient treatment at the Center for Inpatient Forensic Therapy at the Zurich University Hospital of Psychiatry between 1982 and 2016 [with 296 cases being treated after 2000, *n* = 339 (91.6%) male, mean age of M = 34.1, SD = 10.2, also see Table 1]. Data collection was done via retrospective file analysis approved by the Zurich Cantonal Ethics Committee (Ref.-No. KEK-ZH-NR 2014-0480). One hundred seventy-three of these 370 offender patients with SSD had no record of CM, while 197 had experienced one or more of the specified types of CM (emotional or physical neglect, emotional or physical abuse, sexual abuse). Standard treatment of offender patients at this forensic psychiatric hospital include antipsychotic and other medication, psychotherapeutic (mostly cognitive behavioral) interventions, occupational therapy and other social support. File review was executed by a trained psychiatrist with 10% also being coded by a second trained independent rater to ensure inter-rater reliability (Cohens's Kappa of 0.78). Both raters employed directed qualitative content analysis (16) with an objective coding protocol based on an extended set of criteria by Seifert and Leygraf (17–19) and additional parameters adopted from relevant literature. Diagnosis of SSD was based on both the international classification of diseases (ICD) (20) and diagnostic and statistical manual (DSM) (21) of psychiatric disorders. All files examined in the present study contained psychiatric and somatic anamneses, past and present psychiatric inpatient and outpatient reports, legally binding police reports, testimonies in a court of law, court proceedings, reports from social workers, and biannual reports from the nursing and care staff. Due to the significant legal consequences of all this documented material with insufficiencies being challenged by various legal stakeholders, its quality can be considered to be high in Switzerland.

**Table 1:** Descriptive statistics on offender patients with SSD and CM vs. with SSD without CM.

**Variable**	**CM** **experienced**		**No CM** **experienced**	
	**n/N**	**%**	**n/N**	**%**
Mean age at admission (and SD)	32.54	10.12	35.99	10.07
Male gender	179/197	90.9	160/173	92.5
Country of birth Switzerland	107/197	54.3	60/173	34.7
Single (at time of index offense)	167/194	86.1	130/170	76.5
Educational level: Graduation from mandatory schooling (at time of index offense)	62/193	32.1	70/149	47.0
Unemployed (at time of index offense)	156/194	80.4	108/158	68.4
Homeless (at time of index offense)	32/133	24.1	16/97	16.5
Poverty in the patients' childhood/adolescence	76/177	42.9	31/106	29.2
Mean age at first diagnosis of SSD (and SD)	26.39	8.84	30.15	9.29
Suicidal in past	133/194	68.6	99/162	61.1
Suicide attempt in past	77/193	39.9	40/158	25.3
**Substance abuse**				
Alcohol	123/188	65.4	80/139	57.6
Cannabis	136/197	69.0	87/173	50.3
Opiod	65/197	33	38/173	22
Cocaine	80/197	40.6	43/173	24.9
Stimulants/ amphetamines/ecstasy	54/197	27.4	28/173	16.2
Personality disorder present	31/197	15.7	16/171	9.4
Other psychiatric/somatic comorbidity	74/196	37.8	51/169	30.2
Mean IQ score (and SD)	94.72	14.76	90.96	14.3
Mean length of forensic psychiatric inpatient treatment (and SD)	135.97	9.99	86.98	9.79
**Medicaition during current hospitalization**				
Antipsychotics	190/194	97.9	160/166	96.4
Benzodiazepines	31/167	18.6	21/145	14.5
Antidepressants	18/166	10.8	13/145	9.0
Olanzapine equivalents at discharge (and SD)	19.32	13.01	19.14	15.19

*SSD, schizophrenia spectrum disorder; CM, childhood maltreatment; IQ, intelligence quotient; SD, standard deviation*.

### Measures for Subsequent Data Analysis

All variables analyzed in this study are presented in the Supplementary Materials. Childhood was defined according to Swiss law to last from birth until the completion of 18 years of age. CM was defined as to include emotional and physical neglect, emotional and physical abuse and sexual abuse according to definitions provided by the short form of the Childhood Trauma Questionnaire (CTQ-SF) (5) and was rated as being absent or present during file analysis. In addition, the observation of domestic violence during childhood was recorded as absent or present. Before and after forensic psychiatric inpatient treatment, symptoms of SSD were assessed based on all categories of the positive and negative syndrome scale (PANSS) (22) in terms of being absent, being discretely present, or being distinctly present. This data served for computing change in psychopathology during inpatient treatment. An adoption of the CTQ-SF and PANSS for coding seemed adequate, since symptoms of CM and psychopathology were recorded by licensed psychiatrists and clinical psychologists during inpatient treatment with high requirements in professional merit. As a limitation, it may be argued that both the CTQ-SF and the PANSS did not become a standard instrument of psychopathological assessment until after 2003 and 1987, respectively. Psychopathological symptoms before admission to forensic inpatient treatment (at first diagnosis of SSD and in the 12 months prior to the index offense) were coded in broader psychopathological categories also based on the PANSS (see Supplementary Materials). This adjustment was adopted because psychopathology prior to admission to forensic psychiatric inpatient treatment may have been recorded by diagnosing physicians not fully trained in psychiatry in some instances. Previous and index offenses were coded based on common categories of severity. Future prognosis for release from forensic detainment and probability for further criminal behavior was coded based on the last available interdisciplinary assessment of offender patients, which can be expected to be based on the utmost diligence due to the associated legal consequences. These measures were included as a proxy for social functioning of offender patients at the end of inpatient forensic psychiatric treatment as estimated by interdisciplinary professionals. For descriptive statistics, psychiatric medication during forensic inpatient treatment was assessed, including the cumulative daily olanzapine equivalent of all prescribed antipsychotic medication, which was derived by converting dosages based on the classical weighted mean dose method (23), the minimum effective dose method (24) or international experts' consensus (25) in order of availability of conversion factors.

### Statistical Analysis

Latent class analysis (LCA) was used to group offender patients into homogenous subgroups based on the parameters explored, thus creating a new multidimensional discrete latent variable (the “latent” class or subgroup) based on a cross-classification of the observed parameters (26). Based on extant literature, the hypothesis was that offender patients with SSD and CM (in comparison to those with SSD and no CM) would compose a distinct homogenous class with characteristic parameter values (e.g., higher prevalence of all types of CM, certain symptoms of SSD, specific treatment outcome, etc.). In contrast to regression analysis, LCA holds the advantage that groups need not be defined in advance, thus allowing a more explorative and unbiased interpretation of the data set.

Latent class analysis (LCA) was conducted with the poLCA package implemented in R Studio version 1.1.383. Designed for the analysis of multivariate categorical data, LCA categorizes each observation probabilistically into an unobserved (= latent) nominal class, while minimizing the confusion between observations (27). The LCA model is estimated by maximizing the log-likelihood function using the expectation maximization (EM) algorithm (28).

Different numbers of classes (1–4) were evaluated to identify the most parsimonious model with the overall best fit representing the entire data set of 39 variables with 370 observations each. For the given number of classes, each latent class analysis was repeated 500 times with different starting values to avoid local extrema. An LCA model fit is considered stable, if the same solution is found at least twice. Five hundred repetitions were chosen to identify a stable solution with high certainty, but at the same time minimize computation time. In all analyses, each variable was assigned the same prior probability of belonging to a set class, given that no particular expectation regarding classification was available from the literature. For LCA, conditional independence was assumed between the individual variables. Even if this assumption were not fully met, recently it has been shown that error dependencies only add little bias to an independence model (29), particularly if the prevalence is high as was the case with CM (47%) in the current study.

To assess model fit and to compare results with the previous literature, the maximum log-likelihood, the Bayesian information criterion (BIC), Akaike's information criterion (AIC), and entropy were used. Whereas, maximum log-likelihood is exclusively a measure of goodness of model fit, BIC and AIC are measures of parsimony aiming to avoid over-fitting. Entropy is a measure of classification uncertainty (30), with values of > 0.8 indicating a good separation between classes. For a particular number of classes, the model with the lowest log-likelihood was selected. To subsequently compare models between different numbers of classes, information criteria were evaluated. BIC penalizes additional model parameters more strongly than AIC and hence can be considered more conservative in preventing over-fitting. AIC has been reported to overestimate the correct number of components in a finite mixture model (31), whereas BIC performs more adequately (32). For this reason, BIC was prioritized over AIC in selecting the best model fit. scBIC is a sample-size-corrected BIC value being computed for completeness.

## Results

Descriptive statistics on the sample of patients studied are presented in Table 1. Offender patients with SSD and CM did not differ significantly from those without CM in terms of their mean age at first diagnosis of SSD, mean age at admission, gender, mean IQ, antipsychotic or other psychiatric medication or frequency of other somatic or psychiatric comorbidities. Patients with CM were more frequently born in Switzerland, more often grew up in poverty, were less often single, had less frequently graduated mandatory schooling and were more often unemployed and homeless. They had more records of attempted suicide in the past, more illegal substance use (but less abuse of alcohol) and slightly more often a comorbid personality disorder. Their length of stay in forensic psychiatric inpatient treatment was longer than that of patients without CM.

Next, the clinically most relevant results of the LCA are presented. In comparison to the one class-, the two-class and the four-class-model, the three-class model recorded the lowest BIC, indicating best model fit in terms of model complexity and parsimony (see Table 2). The other model fit criteria did not conflict with this solution, nor did clinical theories, once details of the three-class model were examined (next).

**Table 2:** LCA model fit evaluation criteria.

**Number of classes**	**Number of estimated parameters**	**Residual degrees of freedom**	**Maximum log-likelihood**	**AIC**	**BIC**	**scBIC**	**Entropy**	**Number of times solution was found**
1	47	323	−6,835.912	13,765.82	13,949.76	14,079	–	500/500
2	95	275	−6,541.789	13,273.58	13,645.36	13,906	0.8446609	316/500
3	143	227	−6,336.825	12,959.65	**13,519.28**	13,911	0.8937778	200/500
4	191	179	−6,211.936	12,805.87	13,553.35	14,077	0.8278053	37/500

*AIC, Akaike's information criterion; BIC, Bayesian information criterion; entropy, measure of classification uncertainty. Bold value indicates BIC of final model*.

More detailed analysis of the three-class model provides evidence supporting the initial hypothesis, that offender patients with SSD and CM are distinct from those with SSD and no CM not only due to their experiencing of CM, but also due to different characteristics of the other parameters explored. Surprisingly, offender patients with SSD and CM composed two distinct subgroups (classes two and three), while those with SSD and no CM grouped separately (class one). Table 3 presents the clinically most relevant conditional item response probabilities of the three classes with interclass differences above 10% for a subgroup-specific interpretation, as has been suggested elsewhere (33, 34). (Complete results are presented in the Supplementary Materials). Conditional item response probabilities quantify the probability for patients to provide a specific item response depending on the class to which they belong. They allow the comparison of variables amongst the three classes presented (e.g., the probability to be a victim of emotional neglect in class one vs. class two or three). Posterior probabilities, i.e., the probability of a patient with her/ his individual set of item responses belonging to a specific class, can be estimated with Bayes formula (35), but will not be presented here in order to maximize clarity and brevity.

**Table 3:** Conditional item response probabilities of a patient within a particular class to give a specific item response.

**Item**	**Class 1**	**Class 2**	**Class 3**	**Maximum interclass difference**
**Childhood maltreatment**				
**Victim of emotional neglect during childhood**				
No	HIGH	MEDIUM	HIGH	0.23
Yes	LOW	MEDIUM	LOW	0.23
**Victim of emotional abuse during childhood**				
No	VERY HIGH	HIGH	HIGH	0.16
Yes	VERY LOW	LOW	LOW	0.16
**Observer of domestic violence during childhood**				
No	HIGH	MEDIUM	MEDIUM	0.22
Yes	LOW	MEDIUM	MEDIUM	0.22
**Victim of physical neglect during childhood**				
No	MEDIUM	MEDIUM	MEDIUM	0.14
Yes	MEDIUM	MEDIUM	MEDIUM	0.14
**Victim of physical abuse during childhood**				
No	HIGH	MEDIUM	HIGH	0.19
Yes	LOW	MEDIUM	LOW	0.19
**Symptoms at first diagnosis of SSD**				
**Delusions**				
No	MEDIUM	LOW	LOW	0.27
Yes	VERY HIGH	HIGH	HIGH	0.27
**Hallucinations**				
No	LOW	LOW	MEDIUM	0.16
Yes	HIGH	HIGH	MEDIUM	0.16
**Conceptual disorganization**				
No	LOW	HIGH	HIGH	0.34
Yes	HIGH	LOW	LOW	0.34
**Disturbed affect or drive**				
No	VERY LOW	MEDIUM	MEDIUM	0.42
Yes	VERY HIGH	MEDIUM	MEDIUM	0.42
**Negative symptoms**				
No	VERY LOW	MEDIUM	MEDIUM	0.40
Yes	VERY HIGH	MEDIUM	MEDIUM	0.40
**Symptoms prior to index offense**				
**Delusions**				
No	VERY LOW	MEDIUM	LOW	0.35
Yes	VERY HIGH	MEDIUM	HIGH	0.35
**Hallucinations**				
No	LOW	MEDIUM	MEDIUM	0.20
Yes	HIGH	MEDIUM	MEDIUM	0.20
**Conceptual disorganization**				
No	LOW	HIGH	HIGH	0.42
Yes	HIGH	LOW	LOW	0.42
**Disturbed affect or drive**				
No	HIGH	VERY HIGH	VERY HIGH	0.16
Yes	LOW	VERY LOW	VERY LOW	0.16
**Negative symptoms**				
Not present	VERY LOW	VERY LOW	LOW	0.28
Existed discretely	VERY HIGH	MEDIUM	MEDIUM	0.45
Existed distinctly	VERY LOW	LOW	LOW	0.19
**Alogia**				
No	LOW	VERY HIGH	VERY HIGH	0.67
Yes	HIGH	VERY LOW	VERY LOW	0.67
**Blunted affect**				
No	VERY LOW	LOW	HIGH	0.59
Yes	VERY HIGH	HIGH	LOW	0.59
**Apathy**				
No	LOW	VERY HIGH	VERY HIGH	0.78
Yes	HIGH	VERY LOW	VERY LOW	0.78
**Anhedonia**				
No	VERY LOW	MEDIUM	HIGH	0.67
Yes	VERY HIGH	MEDIUM	LOW	0.67
**Social withdrawal**				
No	VERY LOW	MEDIUM	MEDIUM	0.52
Yes	VERY HIGH	MEDIUM	MEDIUM	0.52
**Poor attention**				
No	VERY LOW	HIGH	HIGH	0.58
Yes	VERY HIGH	LOW	LOW	0.58
**History of prior offenses**				
**Previous offenses: assault or property crime with violence**				
No	MEDIUM	HIGH	MEDIUM	0.15
Yes	MEDIUM	LOW	MEDIUM	0.15
**Previous offenses: non-violent crime**				
No	HIGH	HIGH	HIGH	0.14
Yes	LOW	LOW	LOW	0.14
**Index offense leading to forensic hospitalization**				
**Index offense: homicide/attempted homicide**				
No	VERY LOW	VERY LOW	LOW	0.11
Yes	VERY HIGH	VERY HIGH	HIGH	0.11
**Index offense: arson**				
No	HIGH	VERY HIGH	VERY HIGH	0.16
Yes	LOW	VERY LOW	VERY LOW	0.16
**Therapy outcome variables**				
**Psychiatric prognosis on future criminal behavior as estimated at discharge**				
Favorable	LOW	LOW	VERY LOW	0.17
Sufficient	VERY LOW	LOW	LOW	0.10
Doubtful	VERY LOW	LOW	LOW	0.13
Unfavorable	MEDIUM	VERY LOW	LOW	0.34
**Change in PANSS-positive scale from admission to discharge**				
Less symptoms	HIGH	VERY HIGH	LOW	0.54
No change	LOW	VERY LOW	HIGH	0.53
more symptoms	VERY LOW	VERY LOW	VERY LOW	0.01
**Change in PANSS-negative scale from admission to discharge**				
Less symptoms	LOW	HIGH	VERY LOW	0.65
No change	MEDIUM	LOW	HIGH	0.53
More symptoms	VERY LOW	VERY LOW	VERY LOW	0.12
**Change in PANSS-general psychopath. scale from admission to discharge**				
Less symptoms	MEDIUM	VERY HIGH	VERY LOW	0.91
No change	LOW	VERY LOW	VERY HIGH	0.87
More symptoms	VERY LOW	VERY LOW	VERY LOW	0.04
**Change in PANSS-total scale from admission to discharge**				
Less symptoms	HIGH	VERY HIGH	VERY LOW	0.89
No change	LOW	VERY LOW	VERY HIGH	0.88
More symptoms	VERY LOW	VERY LOW	VERY LOW	0.02
Estimated class population share	0.2569	0.2885	0.4546	

*A higher maximal inter-class difference in the conditional item response probabilities observed within the category of a given item indicates a more relevant finding*.

Class one (estimated to comprise 26% of offender patients with SSD) in the final model is characterized by the lowest probability for any CM, with physical neglect being the most frequent CM. By contrast, class two (29% estimated population share) and (to a lesser extent) class three (45% estimated population share) are more probable to experience CM – particularly emotional and physical neglect, as well as physical abuse. At first diagnosis of SSD class one is much more probable than classes two and three to experience delusions, hallucinations, disturbed affect or drive, conceptual disorganization and negative symptoms. Class three is slightly more probable to experience these symptoms than class two, but both are significantly less probable to suffer from such symptoms than class one. In the year prior to the index offense, a similar picture is observable where class one is much more probable to exhibit positive and negative symptoms as well as disturbances of affect and drive than classes two and three. However, now class two is distinctly more probable to experience blunted affect, apathy and anhedonia than class three. In terms of criminal behavior, there is little difference in the probabilities for crimes committed prior to the index offense between classes. Yet for the index offense, class one is clearly more probable to engage in actual or attempted homicide or arson than classes two and three. Except for homicide, class three is slightly more probable than class two to engage in all types of index offenses. There is no difference between classes in their perspective for release from forensic detention, but the prognosis on future criminal behavior is most probable to be favorable for class two and most probable to be unfavorable for class one. It is no surprise then that class two is also most probable to experience a reduction in positive, negative and general psychopathological symptoms, class three is least probable to experience such remission and class one ranges inbetween classes two and three. Negative symptoms were most probable to remain unchanged or even worsen over inpatient treatment in all three classes in comparison to positive symptoms and general psychopathology.

## Discussion

To the authors' best knowledge, this is the first study to explore the role of different types of CM, psychopathology, aspects of criminal behavior and treatment efficacy of offender patients with SSD. Latent class analysis revealed significant differences between three homogenous (latent) patient groups. Among these, class one seems to be least probable to be affected by CM, most probable to experience positive and negative symptoms of SSD, most probable to engage in the most violent of offending and have an average treatment outcome in terms of symptom remission, but the least favorable prognosis for future criminal behavior. Overall, class one seems to be more determined by factors inherent to their SSD, such as positive and negative symptoms of psychopathology, rather than CM related effects. They might represent the “late starters,” a subgroup of offenders suffering from SSD suggested by Hodgins (33).

By contrast, classes two and three were considerably more probable to be affected by CM, particularly physical neglect, physical abuse and emotional neglect. Both classes with SSD and CM, were similarly less probable to experience positive or negative symptoms of SSD and to engage in serious offending, in comparison to those with SSD and no CM. Unexpectedly, however, class two was significantly more probable than class three (and class one) to experience a positive outcome from psychiatric inpatient treatment in terms of a remission of symptoms and favorable prognosis for future offending. The most relevant and distinguishing characteristics of the three classes are summarized in Table 4.

**Table 4:** Overview of the most relevant results in the final 3-class-model.

**Parameter**	**Class 1**	**Class 2**	**Class 3**
CM	–	+	+
Symptoms of SSD	+	–	–
Violent offending	+	–	–
Symptom remission during inpatient treatment	+	+	–
Favorable prognosis on future criminal behavior	–	+	–

*CM, childhood maltreatment; SSD, schizophrenia spectrum disorder*.

Findings on the types of CM most prevalent in offender patients with SSD in the present study confirm prior research, often reporting physical and emotional neglect and sometimes physical abuse to be linked to schizophrenia, but rarely observing any sexual abuse (which may be due to patients not reporting such CM due to shame) (7, 10).

Yet, the presence of two distinct subgroups of offender patients with SSD and CM with one probable to benefit from psychiatric inpatient treatment (class two) while the other (class three) is not probable to benefit, has not been reported so far. This finding assumes particular relevance since the estimated population share of the subgroup with SSD and CM not benefiting during inpatient treatment is almost as big as the other two classes (SSD with and without CM) combined (see estimated class population share in Table 3). The presence of two antithetic subgroups of patients with SSD and CM in terms of treatment outcome, may provide an explanation for the disparity among results from extant research on treatment efficacy in patients with psychosis and CM with only a small pooled odds ratio in a recent meta-analysis (13).

Results (presented here) indicate that patients with SSD and no CM more probably displayed more psychopathology at first diagnosis and prior to their index offense and to be more probable to engage in more violent offending behavior (see Table 3). This is interesting since prior research reported CM to increase odds for SSD (9, 36) and at least double the risk for offending behavior in patients with SSD (4). However, all of these meta-analytic and review based accounts (4, 9, 36) note insufficient differentiation between subtypes of CM (especially emotional abuse vs. physical abuse) and significant variability in defining offending behavior and SSD (or more generally psychosis) in the studies reporting CM to promote SSD or offending behavior. Similarly, Teicher et al. (37) elaborate in their ecophenotype hypothesis that “the myriad (of) possible outcomes of exposure to childhood maltreatment depend on the timing, type, and severity of exposure, plus a host of genetic factors that influence susceptibility and resilience, and an array of protective factors that attenuate risk.”

Future research should explore why some offender patients with SSD and CM benefit from treatment while others do not. The fact that class two is also somewhat more probable than class three to experience blunted affect, apathy and anhedonia prior to committing a crime might hint the role of affective symptoms in this observation. Using network analysis, Isvoranu et al. (7) provided evidence for certain pathways in which specific CM (e.g., physical neglect) is connected to symptoms of general psychopathology (motor retardation), which are then linked to positive and negative symptoms of SSD (blunted affect, anhedonia) – also see introduction. Yet, authors did not consider the role of offending or the possibility for (latent) subgroups of patients with SSD and CM, who may have distinct interconnections between CM, symptoms of SSD and other (confounding) variables, as evidenced in the present study. Personality trait factors may act as confounding variables, even if they do not reach clinical significance to warrant a personality disorder (38). Unfortunately, there was no information available on personality traits in the data analyzed here, unless a personality disorder was diagnosed in addition to SSD. Adding personality disorders to LCA did not provide new insight on the results presented here. Descriptive statistics evidenced patients with CM to have a minimally higher prevalence of personality disorders (15.7 vs. 9.4%) in the sample studied here (see Table 1). Future research should include (subtle) personality traits as a possible confounder. In the present study, descriptive statistics did further enforce the hint on the role of affective symptoms apparent in LCA results, since patients with CM had a higher prevalence of attempted suicide in the past. Similarly, their more frequent use of illegal substances has been observed in prior research and interpreted as an attempt of patients to manage affective symptoms (39).

As a potential limitation, so far, there are no definite guidelines regarding minimal sample size in LCA models. While simulation studies recommended a heuristic number of ≥ 500 (40), many LCA applications did use smaller sample sizes (41). Associated dangers with too small a sample size are the over-extraction of classes, or the diminished detectability of classes with low prevalence. Given that the current study used a sample size of 370 patients, the risks associated with a small sample size need to be considered. Of course, on a cautionary note, the possibility exists that latent class analysis over-extracted classes and thus classes two and three might only reflect dimensional variation within a broader class, with differences in their response to inpatient treatment potentially reflecting fluctuations in the severity of psychotic symptoms over time. In contrast, as there was no underlying theory that suggested a further class, we have no reason to assume that a class was missed because of low prevalence.

Limitations of the current study, not mentioned above, involve the lack of data on (re-)victimization of patients as adults or number of overall traumatic life incidents. This seems unfortunate in light of evidence on a dose response relationship between all types of CM, later traumas and the development of SSD (42–44). However, all data on traumatic experiences are particularly vulnerable to inaccuracies due to unreported trauma (45). Descriptive statistics on the data studied here provide evidence that patients with CM (in comparison to those without CM) were more frequently subject to numerous socio-economic disadvantages, including poverty during childhood, lower levels of education, a higher rate of unemployment and homelessness, despite an average IQ similar to those patients without CM. There is evidence that all of these factors act as stressors increasing the risk for mental illness and criminal offending (46–49). Future research should include such stressors in addition to CM and other trauma in exploring offender patients with SSD and their optimal treatment.

Another limitation pertains to the generalizability of the results presented here. In outpatient settings or inpatient settings in which adherence to therapy is less strictly enforced by legal provisions, additional factors have been reported to mediate poorer therapy outcome in patients with SSD and CM. This includes an avoidant coping style, less compliance to prescribed medication, poor therapeutic alliance and less engagement with treatment services (13). Future studies should include these parameters in analysis in addition to objective measures on social functioning and criminal behavior after release from forensic detention.

Further limitations pertain to retrospective file analysis in general and its specifics in this study. This includes the adoption of the PANSS and Childhood Trauma Questionnaire for coding and coding itself. However, similar to other present-day research (50, 51), retrospective analysis allowed the current study to include a large number of difficult to explore clinically relevant cases with the rare combination of SSD, CM and offending behavior and examine parameters over a prolonged period of time. Future research should review current results using longitudinal and interview based study designs.

## Data Availability Statement

The raw data supporting the conclusions of this article will be made available by the authors, without undue reservation.

## Ethics Statement

The studies involving human participants were reviewed and approved by Kantonale Ethikkommission Zürich Stampfenbachstrasse 121 8090 Zürich. Written informed consent for participation was not required for this study in accordance with the national legislation and the institutional requirements.

## Author Contributions

MG, JK, and SL designed the study and protocol. The survey of the data via questionnaire was preformed independently by JK and SL. All statistical analyses were carried out by MG and supervised by SK. The first draft of the manuscript was done by MG. SL, JK, SK, and SE edited multiple drafts. All authors contributed to the article and approved the submitted version.

## Conflict of Interest

The authors declare that the research was conducted in the absence of any commercial or financial relationships that could be construed as a potential conflict of interest.

## Abbreviations

CM: childhood maltreatment
LCA: latent class analysis
SSD: schizophrenia spectrum disorder.
