# Supplementary material for: Childhood Maltreatment, Psychopathology, and Offending Behavior in Patients With Schizophrenia: A Latent Class Analysis Evidencing Disparities in Inpatient Treatment Outcome
Source: Front Psychiatry. 2021 Jan 28;12:612322. doi: 10.3389/fpsyt.2021.612322 (PMC7875859; doi:10.3389/fpsyt.2021.612322)
Supplement: Supplementary file 1 [file Table_1.DOCX]

SUPPLEMENTARY MATERIAL for: “Childhood maltreatment, psychopathology, and offending behavior in patients with schizophrenia: A latent class analysis evidencing disparities in inpatient treatment outcome”

| **Item** | **Class 1** | **Class 2** | **Class 3** | **Maximum interclass**  **difference** | |
| --- | --- | --- | --- | --- | --- |
| **Childhood maltreatment** |  |  |  |  |  |
| Victim of emotional neglect during childhood |  |  |  |  |  |
| No | 0.7907 | 0.5612 | 0.6888 | 0.23 |  |
| Yes | 0.2093 | 0.4388 | 0.3112 | 0.23 |  |
| Victim of emotional abuse during childhood |  |  |  |  |  |
| No | 0.8608 | 0.7041 | 0.7838 | 0.16 |  |
| Yes | 0.1392 | 0.2959 | 0.2162 | 0.16 |  |
| Observer of domestic violence during childhood |  |  |  |  |  |
| No | 0.7289 | 0.5046 | 0.5401 | 0.22 |  |
| Yes | 0.2711 | 0.4954 | 0.4599 | 0.22 |  |
| Victim of physical neglect during childhood |  |  |  |  |  |
| No | 0.5796 | 0.4423 | 0.4447 | 0.14 |  |
| Yes | 0.4204 | 0.5577 | 0.5553 | 0.14 |  |
| Victim of physical abuse during childhood |  |  |  |  |  |
| No | 0.7430 | 0.5498 | 0.6737 | 0.19 |  |
| Yes | 0.2570 | 0.4502 | 0.3263 | 0.19 |  |
| Victim of sexual abuse during childhood |  |  |  |  |  |
| No | 0.9718 | 0.9401 | 0.9768 | 0.04 |  |
| Yes | 0.0282 | 0.0599 | 0.0232 | 0.04 |  |
| **Symptoms at first diagnosis of SSD** |  |  |  |  |  |
| Delusions |  |  |  |  |  |
| No | 0.0482 | 0.3173 | 0.2584 | 0.27 |  |
| Yes | 0.9518 | 0.6827 | 0.7416 | 0.27 |  |
| Hallucinations |  |  |  |  |  |
| No | 0.2482 | 0.3731 | 0.4042 | 0.16 |  |
| Yes | 0.7518 | 0.6269 | 0.5958 | 0.16 |  |
| Conceptual disorganization |  |  |  |  |  |
| No | 0.3954 | 0.6344 | 0.7319 | 0.34 |  |
| Yes | 0.6046 | 0.3656 | 0.2681 | 0.34 |  |
| Disturbed affect or drive |  |  |  |  |  |
| No | 0.0371 | 0.4141 | 0.4614 | 0.42 |  |
| Yes | 0.9629 | 0.5859 | 0.5386 | 0.42 |  |
| Negative symptoms |  |  |  |  |  |
| No | 0.0845 | 0.4367 | 0.4863 | 0.40 |  |
| Yes | 0.9155 | 0.5633 | 0.5137 | 0.40 |  |
| **Symptoms prior to index offence** |  |  |  |  |  |
| Delusions |  |  |  |  |  |
| No | 0.0601 | 0.4118 | 0.2713 | 0.35 |  |
| Yes | 0.9399 | 0.5882 | 0.7287 | 0.35 |  |
| Hallucinations |  |  |  |  |  |
| No | 0.2990 | 0.4249 | 0.4948 | 0.20 |  |
| Yes | 0.7010 | 0.5751 | 0.5052 | 0.20 |  |
| Conceptual disorganization |  |  |  |  |  |
| No | 0.3539 | 0.7035 | 0.7787 | 0.42 |  |
| Yes | 0.6461 | 0.2965 | 0.2213 | 0.42 |  |
| Disturbed affect or drive |  |  |  |  |  |
| No | 0.7673 | 0.9284 | 0.9085 | 0.16 |  |
| Yes | 0.2327 | 0.0716 | 0.0915 | 0.16 |  |
| Negative symptoms |  |  |  |  |  |
| Not present | 0.0000 | 0.1520 | 0.2848 | 0.28 |  |
| Existed discretely | 0.9065 | 0.5619 | 0.4518 | 0.45 |  |
| Existed distinctly | 0.0935 | 0.2861 | 0.2633 | 0.19 |  |
| Alogia |  |  |  |  |  |
| No | 0.2382 | 0.8724 | 0.9033 | 0.67 |  |
| Yes | 0.7618 | 0.1276 | 0.0967 | 0.67 |  |
| Blunted affect |  |  |  |  |  |
| No | 0.0240 | 0.3603 | 0.6143 | 0.59 |  |
| Yes | 0.9760 | 0.6397 | 0.3857 | 0.59 |  |
| Apathy |  |  |  |  |  |
| No | 0.2071 | 0.8635 | 0.9842 | 0.78 |  |
| Yes | 0.7929 | 0.1365 | 0.0158 | 0.78 |  |
| Anhedonia |  |  |  |  |  |
| No | 0.0443 | 0.5964 | 0.7191 | 0.67 |  |
| Yes | 0.9557 | 0.4036 | 0.2809 | 0.67 |  |
| Social withdrawal |  |  |  |  |  |
| No | 0.0750 | 0.5985 | 0.4188 | 0.52 |  |
| Yes | 0.9250 | 0.4015 | 0.5812 | 0.52 |  |
| Poor attention |  |  |  |  |  |
| No | 0.1636 | 0.7404 | 0.6637 | 0.58 |  |
| Yes | 0.8364 | 0.2596 | 0.3363 | 0.58 |  |
| **History of prior offences** |  |  |  |  |  |
| Previous offences: homicide/attempted homicide |  |  |  |  |  |
| No | 0.9771 | 0.9814 | 0.9699 | 0.01 |  |
| Yes | 0.0229 | 0.0186 | 0.0301 | 0.01 |  |
| Previous offences: assault or property crime with violence |  |  |  |  |  |
| No | 0.4718 | 0.6252 | 0.5104 | 0.15 |  |
| Yes | 0.5282 | 0.3748 | 0.4896 | 0.15 |  |
| Previous offences: rape, sexual assault, or sexual abuse of children |  |  |  |  |  |
| No | 0.9502 | 0.9388 | 0.9776 | 0.04 |  |
| Yes | 0.0498 | 0.0612 | 0.0224 | 0.04 |  |
| Previous offences: arson |  |  |  |  |  |
| No | 0.9722 | 0.9245 | 0.9266 | 0.05 |  |
| Yes | 0.0278 | 0.0755 | 0.0734 | 0.05 |  |
| Previous offences: non-violent crime |  |  |  |  |  |
| No | 0.6515 | 0.7931 | 0.7521 | 0.14 |  |
| Yes | 0.3485 | 0.2069 | 0.2479 | 0.14 |  |
| **Index offence leading to forensic hospitalization** |  |  |  |  |  |
| Index offence: homicide/ attempted homicide |  |  |  |  |  |
| No | 0.1093 | 0.1801 | 0.2169 | 0.11 |  |
| Yes | 0.8907 | 0.8199 | 0.7831 | 0.11 |  |
| Index offence: assault or property crime with violence |  |  |  |  |  |
| No | 0.5386 | 0.6060 | 0.5298 | 0.08 |  |
| Yes | 0.4614 | 0.3940 | 0.4702 | 0.08 |  |
| Index offence: rape, sexual assault, or sexual abuse of children |  |  |  |  |  |
| No | 0.9199 | 0.9029 | 0.8869 | 0.03 |  |
| Yes | 0.0801 | 0.0971 | 0.1131 | 0.03 |  |
| Index offence: arson |  |  |  |  |  |
| No | 0.7931 | 0.9493 | 0.9291 | 0.16 |  |
| Yes | 0.2069 | 0.0507 | 0.0709 | 0.16 |  |
| Index offence: non-violent crime |  |  |  |  |  |
| No | 0.4208 | 0.4691 | 0.4276 | 0.05 |  |
| Yes | 0.5792 | 0.5309 | 0.5724 | 0.05 |  |
| **Therapy outcome variables** |  |  |  |  |  |
| Perspective for release from forensic detainment |  |  |  |  |  |
| 1-5 years | 0.1318 | 0.1723 | 0.1492 | 0.04 |  |
| 6-10 years | 0.0121 | 0.0000 | 0.0071 | 0.01 |  |
| not to be expected | 0.0808 | 0.0429 | 0.0660 | 0.04 |  |
| already released | 0.7753 | 0.7847 | 0.7778 | 0.01 |  |
| Psychiatric prognosis on future criminal behavior as estimated at discharge |  |  |  |  |  |
| favourable | 0.2168 | 0.3452 | 0.1742 | 0.17 |  |
| sufficient | 0.1744 | 0.2747 | 0.2770 | 0.10 |  |
| doubtful | 0.1063 | 0.2189 | 0.2367 | 0.13 |  |
| unfavourable | 0.5025 | 0.1612 | 0.3120 | 0.34 |  |
| Change in PANSS-positive scale from admission to discharge |  |  |  |  |  |
| less symptoms | 0.6845 | 0.8821 | 0.3440 | 0.54 |  |
| no change | 0.3040 | 0.1179 | 0.6500 | 0.53 |  |
| more symptoms | 0.0116 | 0.0000 | 0.0061 | 0.01 |  |
| Change in PANSS-negative scale from admission to discharge |  |  |  |  |  |
| less symptoms | 0.3151 | 0.7721 | 0.1233 | 0.65 |  |
| no change | 0.5822 | 0.2279 | 0.7549 | 0.53 |  |
| more symptoms | 0.1027 | 0.0000 | 0.1218 | 0.12 |  |
| Change in PANSS-general psychopath. scale from admission to discharge |  |  |  |  |  |
| less symptoms | 0.5878 | 0.9854 | 0.0742 | 0.91 |  |
| no change | 0.3891 | 0.0146 | 0.8895 | 0.87 |  |
| more symptoms | 0.0231 | 0.0000 | 0.0363 | 0.04 |  |
| Change in PANSS-total scale from admission to discharge |  |  |  |  |  |
| less symptoms | 0.6252 | 1.0000 | 0.1058 | 0.89 |  |
| no change | 0.3632 | 0.0000 | 0.8760 | 0.88 |  |
| more symptoms | 0.0116 | 0.0000 | 0.0182 | 0.02 |  |
| Estimated class population share | 02569 | 0.2885 | 0.4546 |  |  |
